# Fe(II) and Tannic Acid-Cloaked MOF as Carrier of Artemisinin for Supply of Ferrous Ions to Enhance Treatment of Triple-Negative Breast Cancer

**DOI:** 10.1186/s11671-021-03497-z

**Published:** 2021-02-23

**Authors:** Zihaoran Li, Xinghan Wu, Wenyu Wang, Chengcheng Gai, Weifen Zhang, Wentong Li, Dejun Ding

**Affiliations:** 1 Department of Pathology, Weifang Medical University, Weifang, 261053 China; 2 College of Pharmacology, Weifang Medical University, Weifang, 261053 China

**Keywords:** Artemisinin, Ferroptosis, Triple-negative breast cancer, Metal–organic frameworks, Reactive oxygen species

## Abstract

Suppression of tumor development by inducing ferroptosis may provide a potential remedy for triple-negative breast cancer, which is sensitive to intracellular oxidative imbalance. Recently, artemisinin (ART) and its derivatives have been investigated as potential anticancer agents for the treatment of highly aggressive cancers via the induction of ferroptosis by iron-mediated cleavage of the endoperoxide bridge. Owing to its poor water solubility and limited intracellular iron content, it is challenging for further application in antitumor therapy. Herein, we developed ferrous-supply nano-carrier for ART based on tannic acid (TA) and ferrous ion (Fe(II)) coated on the zeolitic imidazolate framework-8 (ZIF) with ART encapsulated (TA-Fe/ART@ZIF) via coordination-driven self-assembly. Drug release experiments showed that ART was not nearly released in pH 7.4, while 59% ART was released in pH 5.0 after 10 h, demonstrating the excellent pH-triggered release. Meanwhile, a high level of intracellular ROS and MDA, accompanied with decreasing GSH and GPX4, displayed a newly developed nano-drug system displayed markedly enhanced ferroptosis. Compared with monotherapy, in vitro and vivo tumor inhibition experiments demonstrated higher efficiency of tumor suppression of TA-Fe/ART@ZIF. This work provides a novel approach to enhance the potency of ferroptotic nano-medicine and new directions for TBNC therapy.

## Introduction

Ferroptosis, a newly discovered subtype of cell death, could result in accumulation of iron-dependent lipid hydroperoxides (LPO), leading to the damage of the cell structure and integrity [1–3]. Emerging evidence implied that the activation of ferroptosis by several small molecules is an effective approach for tumor suppression in various experimental cancer models and created high expectations for the potential of ferroptosis as a novel anticancer therapy [4–6]. Triple-negative breast cancer (TNBC) is the most aggressive subtype of breast cancer-lacking targeted therapies and often associated with tumor recurrence, distant metastasis, and resistance to therapy [7]. Previous studies have pointed out that xCT cystine/glutamate antiporter is highly expressed in numerous TNBC cells, playing an important role in maintaining the glutathione (GSH) levels and redox balance [8]. Reduction of the intracellular GSH content can render TNBC cells sensitive to ferroptosis, thereby killing tumor cells [8]. Notably, ferroptosis can also bypass the resistance of TNBC to routine programmed apoptosis [9]. Therefore, strategies or drugs based on inducing ferroptosis may have therapeutic potential for the clinical treatment of refractory TNBC.

Artemisinin (ART), a sesquiterpene lactone that contains a peroxide group, was isolated from the traditional Chinese plant *Artemisia annua* and has demonstrated a desirable antitumor activity in multiple cancer cell lines [10, 11]. Increasing evidence displayed that cancer cells contain significantly more intracellular iron pool than normal cells, while iron-mediated cleavage of the endoperoxide bridge allows ART to selectively cause cell death in multiple cancer cell lines [12, 13]. The iron ion-depended antitumor activity has attracted an increasing attention on ART-regulated ferroptosis [13]. Mechanistically, ART can induce lysosomal degradation of ferritin in an autophagy-independent manner, increasing the cellular levels of ferrous ion and sensitizing cells to ferroptosis [11].

However, whether ART induces ferroptosis in TNBC remains unclear. In addition, a series of factors, such as poor water solubility and insufficient availability of intracellular ferrous ions, limit the further application of ART in antitumor therapy [13]. ART nano-complexes are expected to be successfully used as a prospective nano-drug delivery system for ART-based anti-tumor drugs [14–16]. In recent years, metal–organic frameworks (MOFs), a class of porous polymeric material, is attracting attention due to demonstrations of their large pore sizes, high apparent surface areas, and selective uptake of small molecules [17–19]. As a representative of MOF-type materials, the zeolitic imidazolate framework (ZIF-8) is widely used in the development of nano-medicines with characteristics of pH-responsiveness, high drug loading, and good biocompatibility [20–23]. Furthermore, the ability to incorporate an adjustable surface on MOF permits the control of surface properties and its endowment with multifunctionalities [24, 25]. The supramolecular assembly of a metal–phenolic coordination coat on the MOF surface has recently attracted interest owing to the desirable properties, such as stimuli-responsive disassembly, colloidal stability, and biocompatibility [26, 27]. The metal–phenolic coordination materials on the MOF could be an ideal vehicle for delivering the hydrophobic ART.

**Fig. 1:**
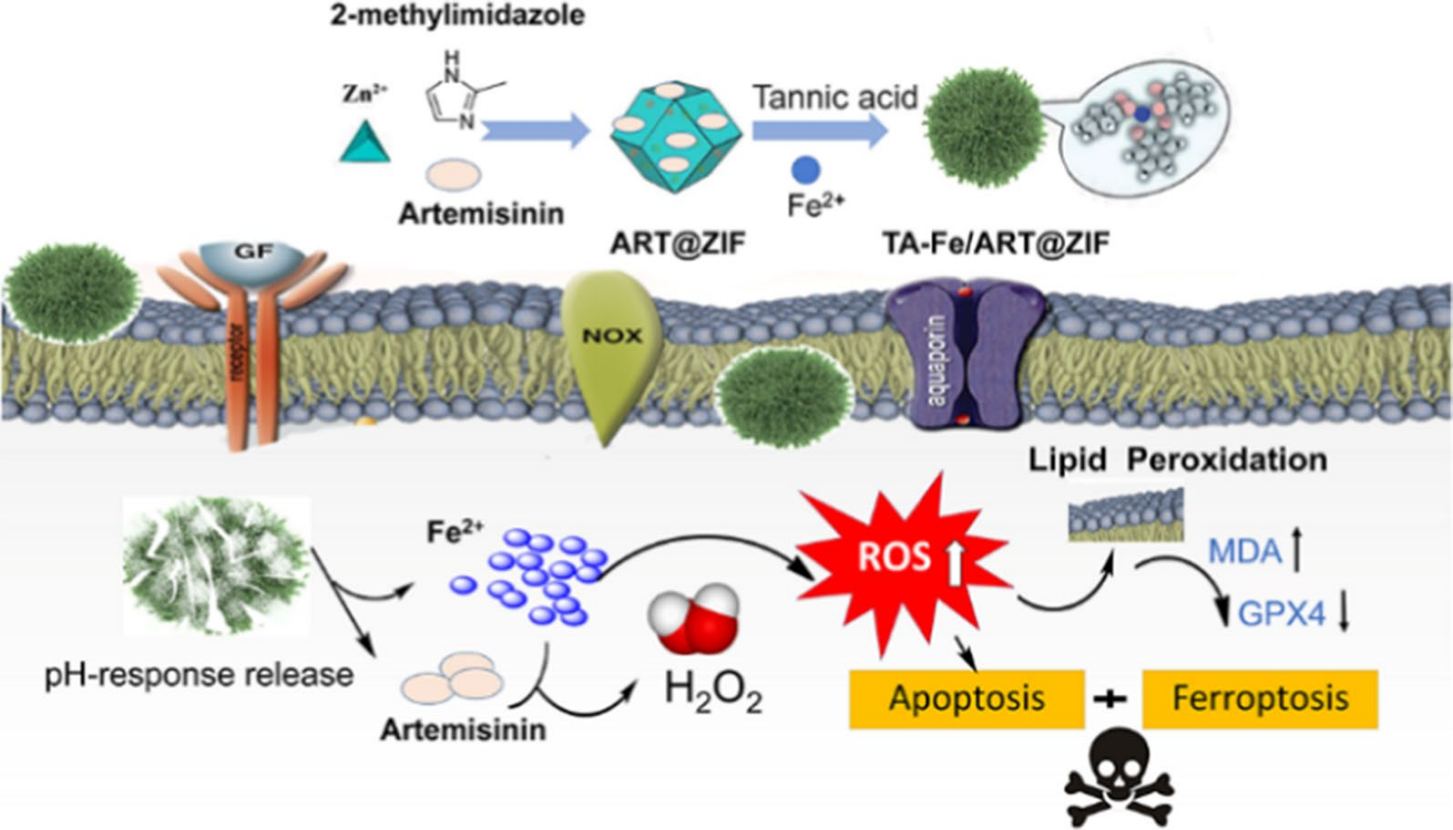
Schematic representation of preparation of TA-Fe/ART@ZIF nanoparticles and the synergistic induction of apoptosis/ferroptosis in tumor cells

Inspired by this, we developed ferrous ion–tannic acid coordination-cloaked ZIF-8 nano-system encapsulating ART (TA-Fe/ART@ZIF) for regulating ferroptosis in TNBC cells, which is demonstrated in Fig. 1. ZIF-8 was selected as a nano-carrier to encapsulate ART owing to its good bio-compatibility and pH-responsive release. The ferrous ion–TA coordination coat was immobilized onto the surface of ART@ZIF for the purpose of dispersion stability and supply of ferrous ions (Fe(II)). Following the internalization of the as-prepared nano-system in cells, acidic degradation of the vehicles would facilitate the release of ART and accumulation of Fe(II). The upregulation of Fe(II) levels in cells would decompose ART into radicals through cleavage of the iron-mediated endoperoxide bridge, markedly enhancing the effects of ferroptosis. Conclusively, the discovery of TA-Fe/ART@ZIF-mediated ferroptosis may offer new perspectives for the development of novel treatments against TNBC.

## Material and Methods

### Reagents

Artemisinin (99%), 2-methylimidazole (98%), zinc nitrate (ZnNO_3_; 98%), Ta (98%), ferrous sulfate (FeSO_4_; 98%), and anhydrous methanol were provided by Aladdin-Reagent Co. Ltd. (Shanghai, China).

### Fabrication of TA-Fe/ART@ZIF Nanoparticles

For the preparation of ART@ZIF nanoparticles, 200 mg of ART was dissolved in 1 mL of anhydrous methanol, and 2 g of 2-methylimidazole (the solvent was 8 mL of absolute methanol) was slowly added to the obtained ART solution. Under magnetic stirring, 0.2 g of zinc nitrate (the solvent was 1 mL of absolute methanol) was slowly added. Finally, the volume of the solution was adjusted to 15 mL and stirred for 10 min to obtain a light white solution. After centrifugation at 10,000 rpm, the sample was washed thrice with methanol. The supernatant was to measure the content of ART, while the precipitant was freeze-dried to obtain the solid state for further use.

For the preparation of TA-Fe/ART@ZIF nanoparticles, TA solution (40 mg/mL in deionized water, 2 mL) was slowly added to the ART@ZIF solution (10 mg/mL, 4 mL). After stirring for 20 min, 5 mg/mL of FeSO_4_ was added slowly to the above solution. After repeated stirring for 30 min, a dark purple solution was obtained. Finally, precipitate was collected by centrifugation at 10,000 rpm. The precipitate was washed three times with deionized water and freeze-dried to obtain the TA-Fe/ART@ZIF for further use.

### Characterization

TEM (JEM-1230; JEOL, Tokyo, Japan) was used to determine the morphological and elemental composition of each part of the nanoparticle. Dynamic light scattering and zeta potential (DLS; Zetasizer Nano system Malvern Instruments, Malvern, UK) were used to evaluate the particle size and electrical stability of the nanoparticles. Fourier transform infrared spectroscopy (VERTEX 70; Bruker, Bremen, Germany) and thermogravimetric analysis were used to analyze the composition of the constituents of the nanoparticles. X-ray photoelectron spectroscopic (XPS) measurements were taken using a PHI 5000 Versa Probe III (Physical Electronics). The elemental composition of the TA-Fe/ART@ZIF material was performed by using EDX (Carl Zeiss Model: Neon-40).

### Measurement of Encapsulation Efficiency and Loading Capacity

High-performance liquid chromatography (HPLC) (Agilent 1200; *Agilent* Technologies, Santa Clara, CA) was used to measure the amount of ART in the supernatant. The drug loading and encapsulation rates of ART can be calculated as follows:

$${\text{Drug}}\,{\text{loading}}\,(\%) = \frac{{{\text{Actual}}\,{\text{amount}}\,{\text{of}}\,{\text{drug}}\,{\text{encapsulated}}\,{\text{in}}\,{\text{NPs}}}}{{{\text{Amount}}\,{\text{of}}\,{\text{NPs}}}} \times 100\%$$

$${\text{Entrapment}}\,{\text{efficiency}}\,\% = \frac{{{\text{Actual}}\,{\text{amount}}\,{\text{of}}\,{\text{drug}}\,{\text{encapsulated}}\,{\text{in}}\,{\text{NPs}}}}{{{\text{Initial}}\,{\text{of}}\,{\text{amount}}\,{\text{of}}\,{\text{drug}}\,{\text{used}}}} \times 100\%$$

### In Vitro Release and pH-Response of TA-Fe/ART@ZIF Nanoparticles

The treated dialysis membrane wrapped with 2 mg of nanoparticles was placed in 50 mL of phosphate-buffered saline (PBS) with pH of 7.4 and 5.0, respectively, and shaken continuously at 37 °C. The solution outside the dialysis membrane was sampled at 15 min, 30 min, 45 min, 1 h, 2 h, 4 h, 6 h, 8 h, and 10 h after the initiation of the experiment. The contents of ART in the buffer solution were measured using HPLC.

### Cell Culture

MDA‐MB‐231 and L929 cell lines were acquired from the American Type Culture Collection (American Type Culture Collection, Manassas, VA, USA). Cells were cultured at 37 °C and 5% CO_2_ humidity in RPMI-1640 medium (Solarbio, Beijing, China), which was supplemented with 10% fetal bovine serum (Cyclone, Utah, USA), 100 µg/mL of sodium pyruvate, penicillin, and streptomycin (Solarbio Beijing, China).

### Cellular Toxicity Test In Vitro

Cell viability was determined using 3-(4, 5-dimethylthiazol-2-yl)-2,5-diphenyltetrazolium bromide (MTT) assay according to literature procedures [28, 29].

MDA-MB-231 cells and L929 cells were cultured in standard cell media in 96-well plates (5,000 cells per well) and incubated in 5% CO_2_ at 37 °C for 24 h. The fluid in the well was discarded, and 100 μL per well of the serum-free medium with PBS and different concentrations of ART, TA-Fe/ZIF, TA-Fe/ART@ZIF, deferoxamine (DFO, MedChemExpress, Shanghai, China), N-benzyloxycarbonyl-Val-Ala-Asp(O-Me) fluoromethyl ketone (Z-VAD-FMK, MedChemExpress, Shanghai, China), and ferrostatin-1 (Fer1, MedChemExpress, Shanghai, China) were added to the 96-well plates. After 48 h, 10 μL of MTT (5 mg/mL) was added and incubated for another 4 h. Finally, an automatic enzyme marker (BioTek Instruments Inc., USA) was used to measure the absorbance in each well. Results were expressed as the percentage of cell viability.

### Calcein-Acetoxymethyl (Calcein-AM) Staining Assay

MDA-MB-231 cells were cultured in 24-well plates (2 × 10^4^ cells per well) and incubated for 24 h. Subsequently, the cells were treated with different concentrations of TA-Fe/ART@ZIF nanoparticles for 24 h. After discarding the medium, the cells were stained with Calcein-AM (Beyotime Biotechnology, Shanghai, China) in the dark at 4 °C for 20 min and observed under a fluorescence-inverted microscope (Olympus, Tokyo, Japan).

### Flow Cytometry for Apoptosis

MDA-MB-231 cells were placed in a six-well plate at a density of 2.5 × 10^5^ cells per well under the same conditions. After treatment with PBS, ART, TA-Fe/ZIF, and TA-Fe/ART@ZIF were applied for 24 h. Subsequently, the cells were centrifuged and collected from the six-well plate. After propidium iodide and Annexin-V double staining (Annexin V‐FITC Kit; Beckman Coulter, Marseille, France), flow cytometry was used to detect.

### In vitro Reactive Oxygen Species (ROS) Determination Assay

The ROS content in cells was performed using a ROS fluorescence probe (dichloro-dihydro-fluorescein diacetate (DCFH-DA, Beyotime Biotechnology, Shanghai, China). MDA-MB-231 cells were cultured in six-well plates (2.5 × 10^5^ cells per well) and incubated in 5% CO_2_ at 37 °C for 24 h. The fluid from the wells was discarded, and cells were treated as follows: blank control group (serum-free medium), positive control group, and experimental group (different concentrations of nanoparticles). Following incubation for 8 h at 37 °C, 0.1% DCFH-DA was added to each well and the cells were incubated for 30 min. Cells unresponsive to DCFH-DA were removed with PBS and observed under a fluorescence-inverted microscope (Olympus, Tokyo, Japan).

### Malondialdehyde (MDA) and GSH Content Determination

The MDA assay kit (TBA method; Jiancheng Bioengineering, Nanjing, China) and GSH assay kit (Beyotime Biotechnology, Shanghai, China) were used to measure the intracellular levels of MDA and GSH. After treatment with PBS, ART, TA-Fe/ZIF, and TA-Fe/ART@ZIF, MDA-MB-231 cells were collected and counted. The intracellular content of MDA and GSH was determined according to the instructions provided in the kits.

### Western Blot Analysis

The MDA-MB-231 cells, which treated with different nanoparticles, were lysed with RIPA lysis buffer. After the protein concentration was determined, the proteins of different samples were separated using 10% SDS-PAGE gel and transferred to nitrocellulose membrane. The nitrocellulose membrane that loaded with sample proteins was blocked by 0.5% BSA protein solution for 1 h, and the nitrocellulose membrane and the primary antibodies were incubated for 24 h at 4 °C. We rinsed the primary antibodies from the nitrocellulose membrane with TBST and continued to place it with the corresponding secondary antibodies at ordinary temperature for 2 h. After washing off the secondary antibodies on the in cellulose membrane, the nitrocellulose membrane was used a chemiluminescent solution and observed under the gel imaging system.

### In Vivo Antitumor Experiment

All animal experiments were approved by the Ethics Committee of Weifang Medical University. Healthy female nude mice (age: 4 weeks; weight: 13–17 g; Vital River, Beijing, China) were administrated with 5 × 10^6^ MDA-MB-231 cells in of 150 µl of phosphate-buffered saline. When the tumor volume increased to 70–120 mm^3^, the mice were randomly divided into four groups. Each group of mice was separately treated with PBS, ART (20 mg/kg), TA-Fe/ZIF (80 mg/kg), and TA-Fe/ART@ZIF (100 mg/kg). Each mouse was treated by intraperitoneal injection every three days. After 14 days, all mice were killed. The tumor tissues and important organs were collected for hematoxylin and eosin staining.

### Statistical Analysis

All experiments were repeated at least thrice. All data were statistically analyzed using the SPSS version 22.0 software (IBM Corp., Armonk, USA). The results were expressed as the mean ± standard deviation. *P* values < 0.05 denoted statistical significance.

## Results and Discussion

### Characterizations of TA-Fe/ART@ZIF Nanoparticles

**Fig. 2:**
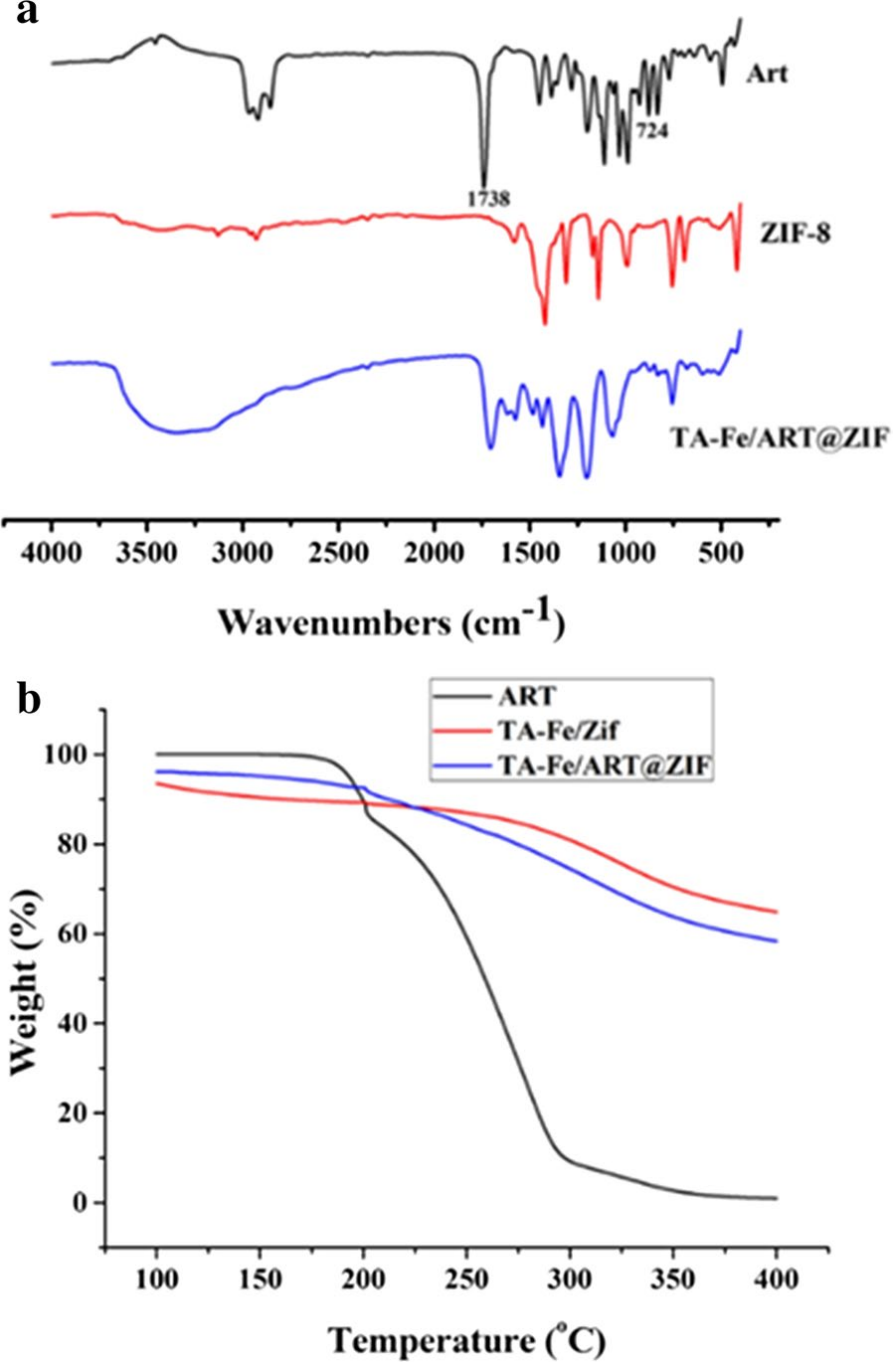
**a** FTIR of ART, ZIF-8, and TA-Fe/ART@ZIF; **b** Thermogravimetric analysis (TGA) of ART, TA-Fe/ZIF, and TA-Fe/ART@ZIF

First, the ART@ZIF nanoparticles were synthesized at room temperature from methanol, zinc acetate, 2-methylimidazole, and ART according to literature procedures [30]. Stable metal-polyphenol supramolecular films were then rapidly formed around the ART@ZIF templates by vortexing TA and ferrous ions. Compared to the reported magnetic nanoparticles [31–33], the TA-Fe/ART@ZIF was prepared via the self-assembly method formatting TA-Fe membrane on the surface of MOF without involved harsh chemical or hydrothermal reaction. More importantly, the TA-Fe/ART@ZIF nano-carrier can be triggered by low pH, to release ART and Fe(II), which is further catalyzed by the endoperoxide of ART to generate C-centered free radicals, markedly enhanced ferroptosis. The encapsulation efficiency, which was measured by HPLC using the supernatant from the first centrifugation, was 66.7%. The supernatant was obtained by the TA-Fe/ART@ZIF nanoparticles, and the calculated drug load of ART was 11.4%. According to FTIR spectroscopy (Fig. 2a), characteristic absorption peaks of ART, namely the carbonyl bond at 1,738 cm^−1^ and the peroxy-bridge at 724 cm^−1^ [34], were observed in TA-Fe/ART@ZIF nanoparticles, indicating that ART was successfully encapsulated in the nanoparticles. Next, the results of the thermogravimetric analysis revealed that ART completely abolished when the temperature was increased to approximately 400 °C (Fig. 2b). Compared with the TA-Fe/ZIF nanoparticles, it was found that ART accounts for 7.1% of the total weight of the TA-Fe/ART@ZIF nanoparticles, which is basically consistent with the results of the HPLC analysis.

**Fig. 3:**
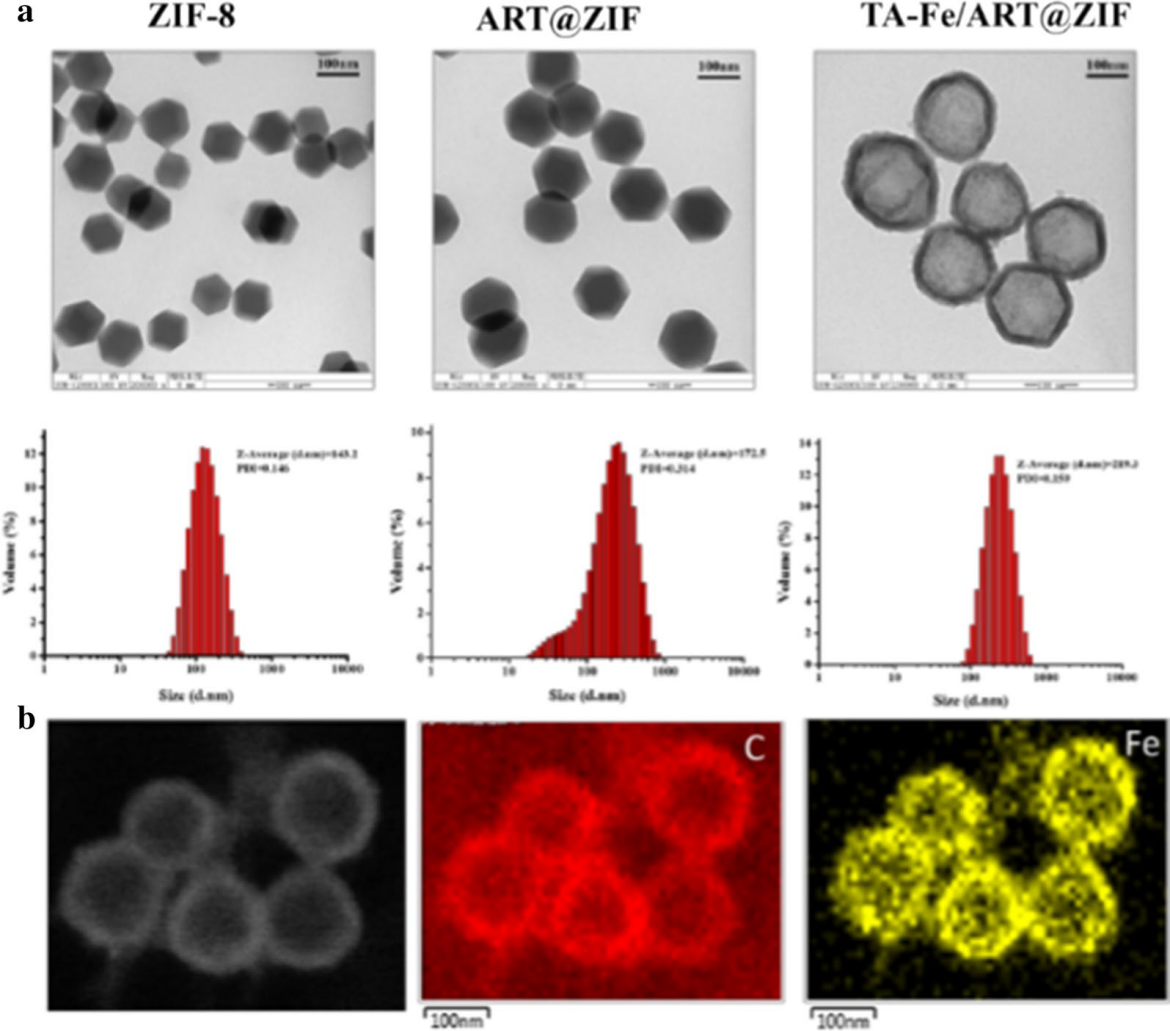
**a** TEM images and size distribution of ZIF-8, ART@ZIF, and TA-Fe/ ART@ZIF nanoparticles. Scale bar: 100 nm; **b** distribution of carbon and iron element in TA-Fe/ ART@ZIF nanoparticles. Scale bar: 100 nm

The results of TEM showed that ZIF-8 and ART@ZIF exhibited a similar uniform hexagon configuration, and the particle size distribution was determined at approximately 100 nm (Fig. 3a, b). TA-Fe/ART@ZIF nanoparticles exhibited a spherical configuration, and the particle size distribution was 150 nm. Compared with complete ART@ZIF, TA-Fe/ART@ZIF coated with Fe(II) and TA demonstrated an obvious conventional core–shell structure, and the size of the TA-Fe membrane was approximately 30 nm (Fig. 3a). Moreover, we performed area-elemental mapping analysis of the formed nanoparticles. The area-elemental mapping confirmed that the periphery of TA-Fe/ART@ZIF nanoparticles was encircled by Fe element, indicating that Fe(II) and TA were successfully cloaked (Fig. 3b). XPS measurements were taken to investigate surface elemental composition and interaction in TA-Fe/ART@ZIF composite. The wide-scan XPS spectrum of TA-Fe/ART@ZIF is depicted in Additional file 1: Fig. 1s. The major peaks appearing at around 285, 408, 531, 737, and 1036 eV were related to C 1s, N 1s, O 1s, Fe 2p, and Zn 2p, respectively, demonstrating the formation of TA-Fe/ART@ZIF nanocomposite. Furthermore, EDS mapping images of the TA-Fe/ART@ZIF are shown in Additional file 1: Fig. 2s. It is found clearly that the peaks corresponding to elements such as carbon (C), nitrogen (N), oxygen (O), iron (Fe), and zinc (Zn), are in good agreement with the XPS spectrum. By increasing the amount of Fe(II), we found that the hydrodynamic diameter of TA-Fe/ART@ZIF nanoparticles was increased (Additional file 1: Fig. 3s). To further confirm the coating, we measured the zeta potential of various nanoparticles. The formation TA–Fe(II) layer on ART@ZIF particles shifted the surface zeta from + 21 mV potential to − 19.5 mV due to the acidic nature of TA (Additional file 1: Fig. 4s). As previous literature reported [30], the abundant polyphenol groups in TA structure not only endow the substrate coating ability, but also are capable of coordinating with transition metal ions (Fe, Zn) to form metal–phenolic complex. When Zn^2+^ and Hmim are mixed to form a solution, a mass of Zn^2+^ ions would coordinate at the surface of ART@ZIF particles; thus, zeta potential of ART@ZIF is + 21 mV. The phenolic groups of TA (pKa 8.5) were deprotonated negatively and could therefore interact with the positively charged Zn^2+^, promoting the TA–Fe film growth on ART@ZIF surface. As drug delivery favors carriers, the stability is an important factor for their medicinal application. The stability of the nanoparticles was demonstrated by dispersing in PBS for one week. As shown in Additional file 1: Fig. 5s, the particle size presented desirable size and stability from time course of study.

**Fig. 4:**
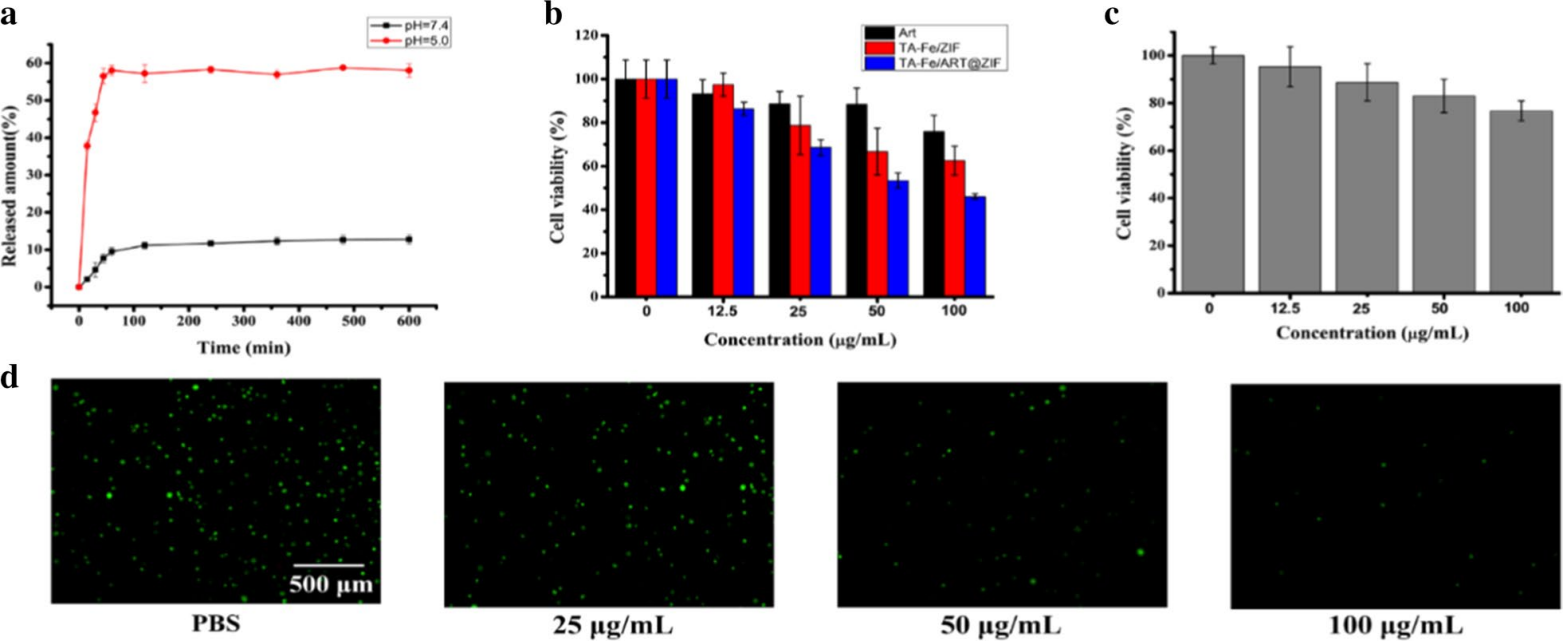
**a** In vitro release of ART from TA-Fe/ ART@ZIF nanoparticles in pH 7.4 and 5.0. **b** The cytotoxicity of ART, TA-Fe/ZIF, and TA-Fe/ART@ZIF nanoparticles in MDA-MB-231 cells at 0, 12.5, 25, 50, and 100 μg/mL. **c** Viability of L929 cells treated with TA-Fe/ART@ZIF at different concentrations. **d** Calcein-AM staining of MDA-MB-231 cells treated with TA-Fe/ART@ZIF nanoparticles at concentrations of 0, 25, 50, and 100 μg/mL

**Fig. 5:**
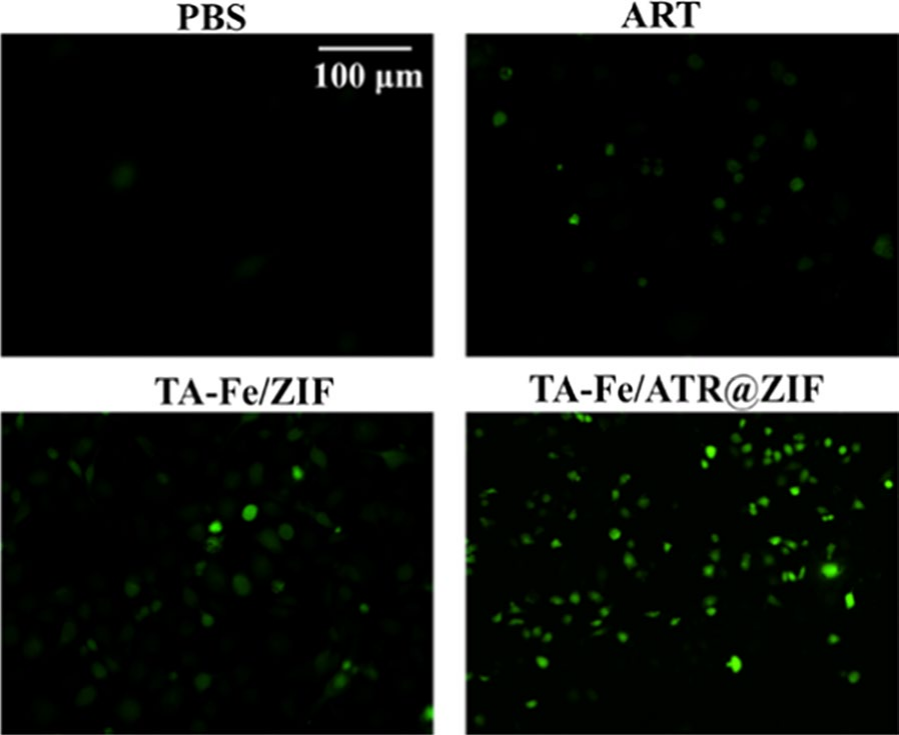
ROS production detected by fluorescence of DCFH-DA in MDA-MB-231 cells treated with ART, TA-Fe/ZIF, and TA-Fe/ART@ZIF nanoparticles

### Release and Cytotoxicity of TA-Fe/ART@ZIF In Vitro

Next, in order to study whether TA-Fe/ART@ZIF had pH-responsive decomposition behavior, we examined the release of ART from nanoparticles under neutral and acidic conditions. As shown in Fig. 4a, in the acidic conditions, the nanoparticles can rapidly release approximately 58% of ART within 1 h. However, under neutral conditions, the nanoparticles can only slowly release a minute amount of ART. In addition, during the treatment of the external solution of the dialysis membrane with sodium hydroxide, the solution of the pH = 5.0 group was reacted with sodium hydroxide to produce a considerable white precipitate. However, this phenomenon was not observed in the pH = 7.4 group.

According to our hypothesis, the white precipitate is a zinc hydroxide precipitate formed by the dissociation of zinc ions and alkali from the nanoparticles under acidic conditions. Collectively, this evidence successfully shows that our nanoparticles have the ability to dissociate under acidic conditions.

In the design of TA-Fe/ART@ZIF nano-system, the TA-Fe(II) and ZIF structures are ablated to release the encased ART and Fe(II) under the acidic conditions of the tumor microenvironment. The upregulation of Fe(II) levels in cells would decompose ART into radicals through cleavage of the iron-mediated endoperoxide bridge, markedly enhancing the effects of ferroptosis. Accordingly, MTT assays were conducted to study the cytotoxicity of nano-system to MDA-MB-231 cells and L929 cells. Compared with ART, TA-Fe/ ART@ZIF nanoparticles showed greater cytotoxicity to MDA-MB-231 cells (Fig. 4b) and low cytotoxicity to normal cells (Fig. 4c). TA-Fe/ ART@ZIF nanoparticles inhibited the activity of MDA-MB-231 cells by 53.9%, while ART at the same concentration inhibited only 24.1% of the whole cells. The experimental results of calcein-AM staining confirmed that the quantity of viable MDA-MB-231 cells gradually descended with the increasing concentration of TA-Fe/ ART@ZIF nanoparticles (Fig. 4d).

### TA-Fe/ART@ZIF Enhanced ROS Generation in MDA-MB-231 Cells

ART is known to exert its anticancer activity via generation of ROS produced by iron-mediated cleavage of the endoperoxide bridge [35, 36]. Therefore, we have examined the efficiency of TA-Fe/ ART@ZIF to induce ROS generation by the 2′,7′-dichlorodihydrofluorescein diacetate (DCHF-DA) probe [37]. As shown in Fig. 5, the slightly stronger fluorescence signal was observed in cells treated with ART and TA-Fe/ZIF compared with untreated groups, indicating that the ART and Fe(II) ions could induce ROS generation. By contrast, strong fluorescence signal exposure to TA-Fe/ ART@ZIF nano-drug is found in cells as an evidence of desired ROS yield, which is generated by cleavage of Fe(II)-mediated endoperoxide of ART. The as-prepared nano-system in cells would be degradated and make accumulation of Fe(II), remarkably enhanced ROS generation. The anticancer effect of ART and its derivatives have been attributed to their ability to induce apoptosis by various cellular processes, ranging from DNA damage response, the lysosome-mediated catabolic process macroautophagy, and oxidative stress [13, 35, 38]. And it was also reported that the iron-mediated cleavage of the endoperoxide bridge in ART could affect the intracellular oxidative balance to ferroptosis in many types of cancer cells [11]. This induction of ferroptosis based on oxidative imbalance can be further amplified with the addition of Fe(II). While Fe(II) activates ART, it can also react with intracellular hydrogen peroxide to generate hydroxyl free radicals through the Fenton reaction, which enhances the apoptosis-inducing effect of ART in tumor cells.

### TA-Fe/ART@ZIF Induced Apoptosis and Ferroptosis in MDA-MB-231 Cells

**Fig. 6:**
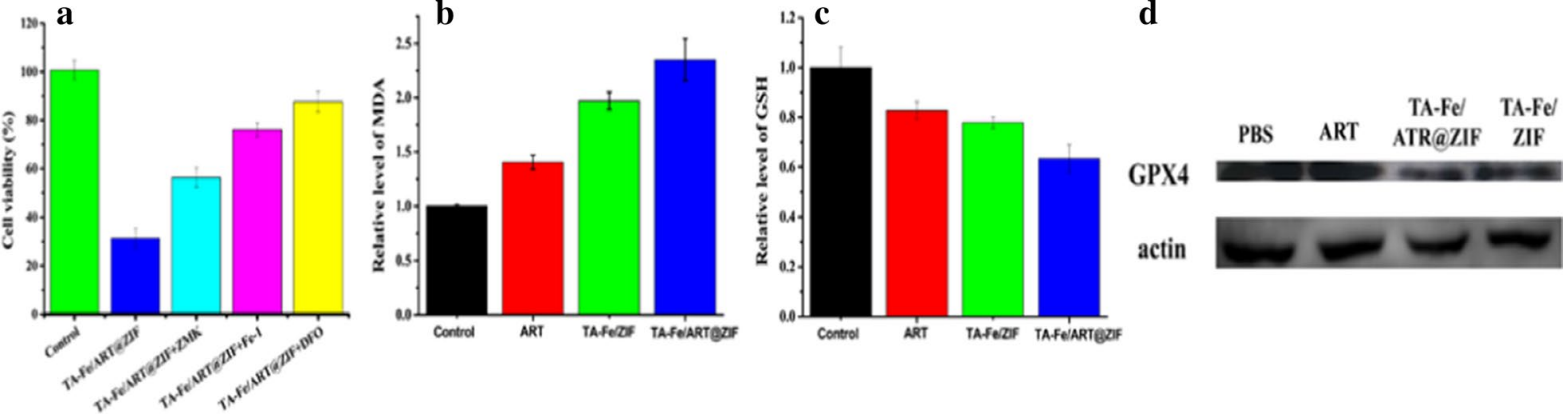
**a** Inhibitors DFO (deferoxamine), Fer-1 (Ferrostatin-1) and Z-VAD-FMK rescued the viability of MDA-MB-231 cells under treatment with 100 μg/mL TA-Fe/ART@ZIF nanoparticles, respectively. **b** ART, TA-Fe/ZIF, and TA-Fe/ART@ZIF nanoparticles contributed to excessive MDA in MDA-MB-231 cells. **c** ART, TA-Fe/ZIF, and TA-Fe/ART@ZIF nanoparticles consumed intracellular GSH. **d** The expression levels of GPX4 proteins in MDA‐MB‐231 cells treated with ART, TA-Fe/ZIF, and TA-Fe/ART@ZIF nanoparticles

Meanwhile, to investigate cell death and role of Fe(II), we utilized iron chelator deferoxamine, apoptosis inhibitor Z-VAD-FMK, and ferroptosis inhibitor ferrostatin-1 to rescue these cells. As predicted, deferoxamine, a scavenger of Fe(II), could obviously block cell death, suggesting the important role of Fe(II). Moreover, the survival rate of MDA-MB-231 cells was significantly improved by apoptosis and ferroptosis inhibitor from 31.4% to 56.3% and 76.0%, respectively (Fig. 6a), highlighting the importance of apoptosis and ferroptosis in TA-Fe/ART@ZIF nanoparticles mediated the cell death. In addition, we employed Annexin V- FITC-based assay through flow cytometry, to quantitatively determine the degree of apoptosis. The results state that TA-Fe/ART@ZIF nanoparticles can induce apoptosis in 21.8% of MDA-MB-231 cells (Additional file 1: Fig. 6s). This percentage was higher than that recorded for ART, implying that apoptosis is involved in the TA-Fe/ART@ZIF nanoparticles mediated, but it is not the main cause.

A common feature of ferroptosis is endogenous lipid peroxidation [39]. MDA, a product of lipid peroxidation [40], was investigated to assess the degree of ferroptosis. Results showed TA-Fe/ART@ZIF nanoparticles had approximately 2.5 times higher MDA levels than control group (Fig. 6b). This was presumed due to the presence nanocarrier-enriched Fe(II) and the corresponding elevation of lipid radicals levels. As one of the major antioxidant components in the cells, intracellular GSH is decreased accompanied with ferroptosis [41]. Considering the import role of GSH in ferroptosis, we evaluated intracellular GSH level after treatment with TA-Fe/ART@ZIF nanoparticles. GSH was decreased significantly compared to vehicle-treated cells (Fig. 6c). This provided strong evidence for the depletion of GSH and oxidation imbalance, which was centered on the Fe(II)-mediated activation of ART by TA-Fe/ART@ZIF nanoparticles. Next, western blotting was performed to understand the impact of TA-Fe/ART@ZIF nanoparticles on GPX4 activity [42]. We observed that TA-Fe/ART@ZIF nanoparticles caused a more significantly inhibition of GPX4 activity than ART (Fig. 6d). These data also agreed well with the highest extent of GSH depletion.

### In Vivo Antitumor Experiment

**Fig. 7:**
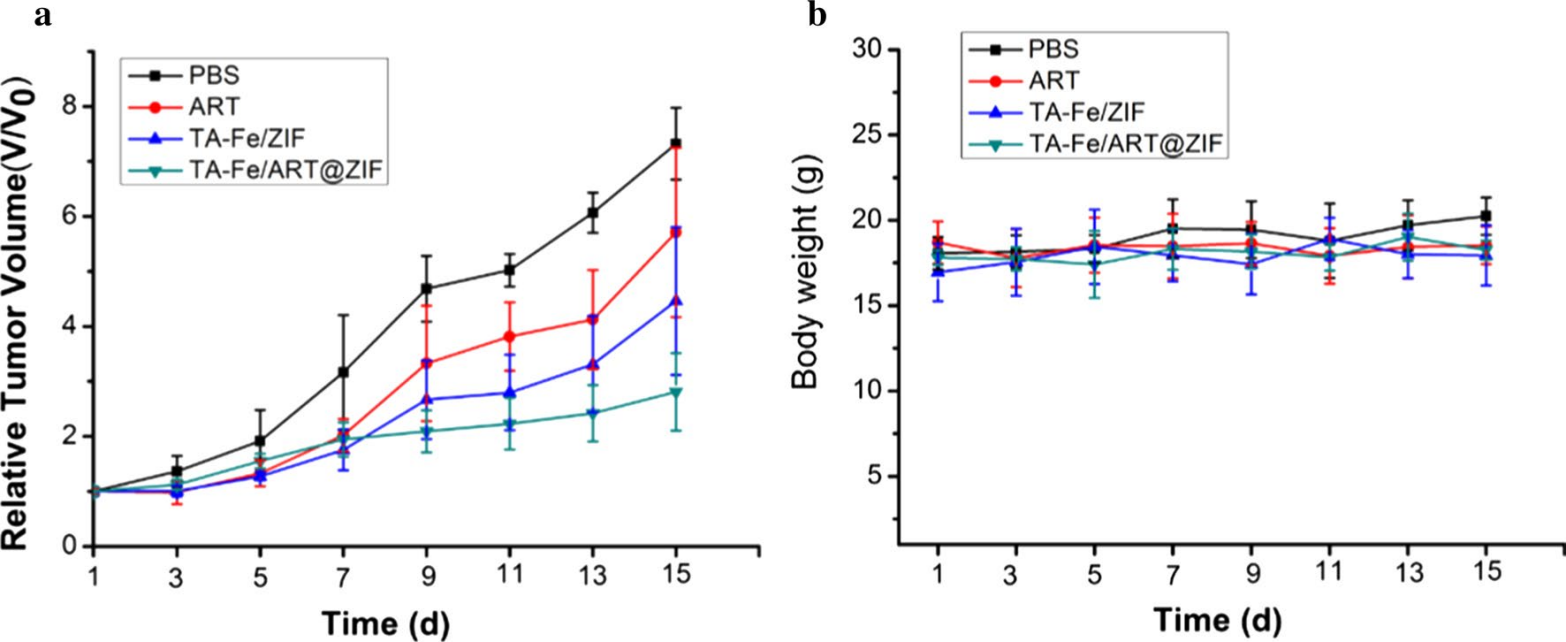
**a** Relative tumor volume after treated with treated with PBS, ART, TA-Fe/ZIF, and TA-Fe/ART@ZIF nanoparticles. **b** Relative mice body weight of various groups

**Fig. 8:**
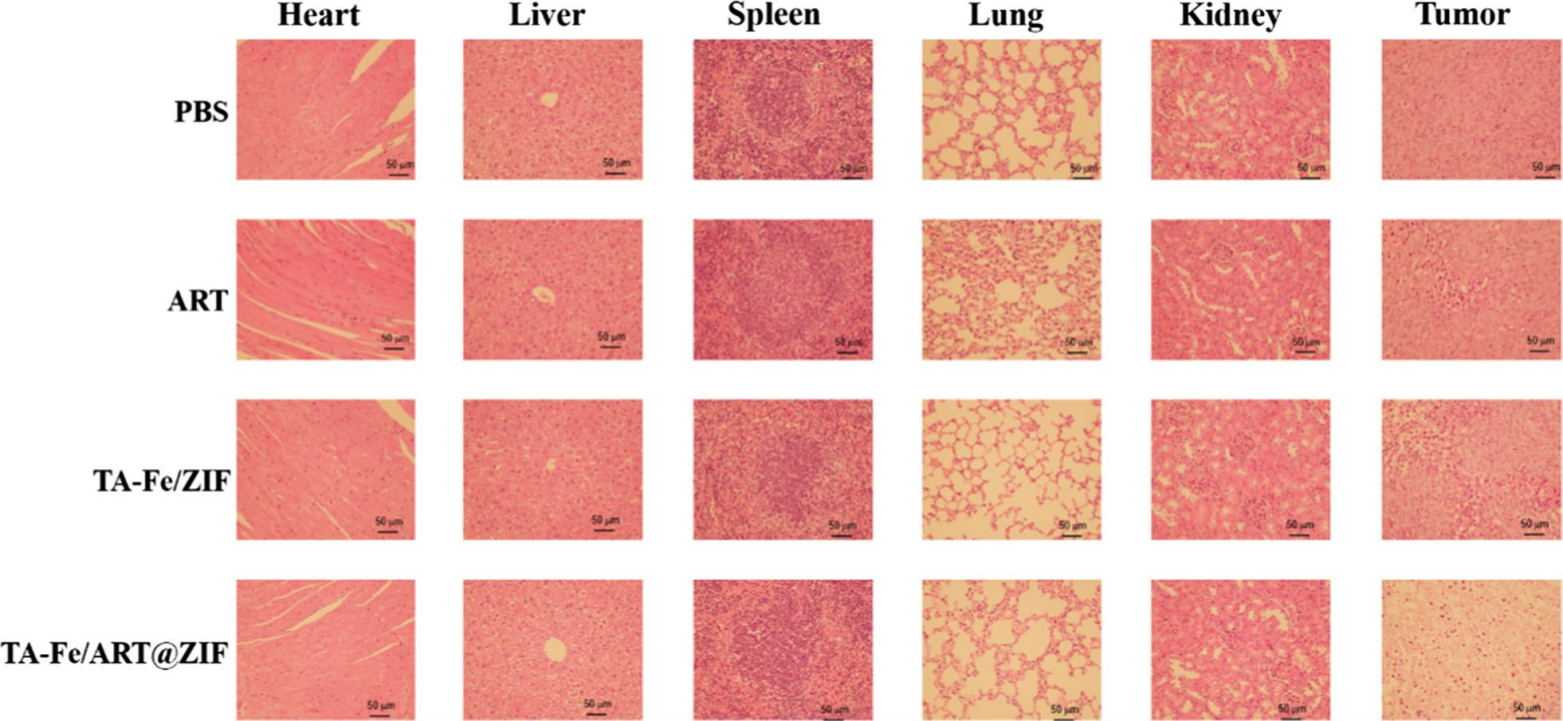
H&E stains of organs and tumors from various mice groups. Scale bar: 50 μm

We evaluated the antitumor efficacy of our nanoparticles to MDA-MB-231 cells in vivo by the inhibition of subcutaneous xenograft tumors in tumor-bearing nude mice. During the 14-day treatment, relative to other groups, TA-Fe/ART@ZIF nanoparticles can significantly inhibit the growth of MDA-MB-231 xenograft tumors as the result of abundant ROS generation through Fe(II)-ART reaction (Fig. 7a). In addition, there was no significant diversity of body weight in all experimental groups (Fig. 7b), manifesting that the TA-Fe/ART@ZIF had negligible side effects. To further study the therapeutic effect, the tumor and other normal tissues were examined by H&E staining after being treated for 14 d. As shown in Fig. 8, TA-Fe/ART@ZIF caused the largest region of cell death in the tumor tissue, while there was no obvious damage and apoptosis in the normal tissues. Overall, these results demonstrated that the TA-Fe/ART@ZIF-mediated therapy not only led to anticancer efficacy, but also have a low level of acute systematic toxicity in the SFT-MB-mediated therapy.

## Conclusion

In summary, ferroptosis provides a potential remedy for TNBC treatments, since it has exclusive advantages to overcome inevitable barriers of the currently prevalent therapy. Here, we designed a ferrous-supply nanocarrier for ART based on TA–Fe(II) coated on the ZIF nanoparticles with ART encapsulated via coordination-driven self-assembly for enhanced ferroptosis. The nano-carrier could be dissolved in the weak acidic microenvironment to release ART and Fe(II), which is further caused a high level of intracellular ROS and MDA, accompanied with decreasing of GSH and GPX4, leading to potent tumor growth inhibition and anticancer efficacy in vitro and in vivo. This work provides a novel approach to enhance the potency of ferroptotic nano-medicine and new directions for TBNC therapy. Biocompatibility and comprehensive mechanisms of TA-Fe/ART@ZIF-induced ferroptosis should be ensured for further investigation.

## Supplementary Information

**Additional file 1: Fig. 1s**. X-ray photoelectron spectroscopic of TA-Fe/ART@ZIF.** Fig. 2s**. EDS spectrum of TA-Fe/ART@ZIF.** Fig. 3s**. Hydrodynamic size of ZIF-8, ZIF-ART and TA-Fe/ART@ZIF in different raw material proportions.** Fig. 4s**. Zeta potential of ZIF-8, ZIF-ART and TA-Fe/ART@ZIF in different raw material proportions.** Fig. 5s**. Time course of size distribution of TA-Fe/ART@ZIF nanoparticles.** Fig. 6s**. ART, TA-Fe/ZIF and TA-Fe/ART@ZIF nanoparticles induced apoptosis in MDA-MB-231 cells detected by flow cytometry.

## Data Availability

All data are fully available without restriction.

## Abbreviations

MOF: Metal–organic frameworks
TNBC: Triple-negative breast cancer
ART: Artemisinin
TA: Tannic acid
ZIF: Zeolitic imidazolate framework-8
TA-Fe/ART@ZIF: Tannic acid and ferrous ion coated on the zeolitic imidazolate framework-8 with artemisinin encapsulated
ROS: Reactive oxygen species
LPO: Lipid hydroperoxides
GSH: Glutathione
TEM: Transmission electron microscopy
FTIR: Fourier transform infrared spectroscopy
HPLC: High-performance liquid chromatography
PBS: Phosphate-buffered saline
MTT: 3-(4,5-Dimethylthiazol-2-yl)-2,5-diphenyltetrazolium bromide
DFO: Deferoxamine
Fer-1: Ferrostatin-1
Z-VAD-FMK: N-Benzyloxycarbonyl-Val-Ala-Asp(O-Me) fluoromethyl ketone
Calcein-AM: Calcein-acetoxymethyl
DCFH-DA: Dichloro-dihydro-fluorescein diacetate
MDA: Malondialdehyde
BSA: Bovine serum albumin
TBST: Tris-buffered saline Tween
SDS-PAGE: Polyacrylamide gel electrophoresis
GPX4: Glutathione peroxidase 4
