# Supplementary material for: Fe(II) and Tannic Acid-Cloaked MOF as Carrier of Artemisinin for Supply of Ferrous Ions to Enhance Treatment of Triple-Negative Breast Cancer
Source: Nanoscale Res Lett. 2021 Feb 23;16:37. doi: 10.1186/s11671-021-03497-z (PMC7902752; doi:10.1186/s11671-021-03497-z)
Supplement: Supplementary file 1 — Additional file 1: Fig. 1s. X-ray photoelectron spectroscopic of TA-Fe/ART@ZIF. Fig. 2s. EDS spectrum of TA-Fe/ART@ZIF. Fig. 3s. Hydrodynamic size of ZIF-8, ZIF-ART and TA-Fe/ART@ZIF in different raw material proportions. Fig. 4s. Zeta potential of ZIF-8, ZIF-ART and TA-Fe/ART@ZIF in different raw material proportions. Fig. 5s. Time course of size distribution of TA-Fe/ART@ZIF nanoparticles. Fig. 6s. ART, TA-Fe/ZIF and TA-Fe/ART@ZIF nanoparticles induced apoptosis in MDA-MB-231 cells detected by flow cytometry. [file 11671_2021_3497_MOESM1_ESM.docx]

**Supporting information**

Fe(II) and tannic acid-cloaked MOFs as carrier of artemisinin for supply of ferrous ions to enhance triple-negative breast cancer treatment

Zihaoran Li ^a,1^, Xinghan Wu ^a,1^, Wenyu Wang ^b^, Chengcheng Gai ^a^, Weifen Zhang ^b^, Wentong Li ^a^ ^[[1]](#footnote-1)^*, Dejun Ding ^b^ ^[[2]](#footnote-2)^*

^a^ Department of Pathology, Weifang Medical University, Weifang 261053, China

^b^ College of Pharmacology, Weifang Medical University, Weifang 261053, China

^1^These authors contributed equally to this work.





**Fig. 1s.**  X-ray photoelectron spectroscopic of TA-Fe/ART@ZIF





**Fig. 2s.**  EDS spectrum of TA-Fe/ART@ZIF.

**

**

**Fig. 3s.** Hydrodynamic size of ZIF-8, ZIF-ART and TA-Fe/ART@ZIF in different raw material proportions.

**

**

**Fig. 4s.** Zeta potential of ZIF-8, ZIF-ART and TA-Fe/ART@ZIF in different raw material proportions.

**

**

**Fig. 5s.** Time course of size distribution of TA-Fe/ART@ZIF nanoparticles.

**
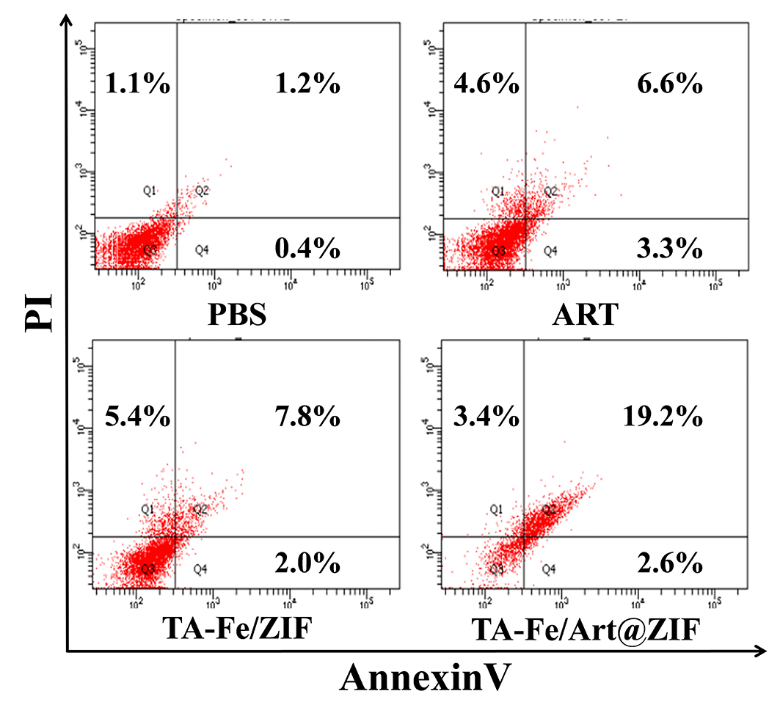
**

**Fig. 6s.** ART, TA-Fe/ZIF and TA-Fe/ART@ZIF nanoparticles induced apoptosis in MDA-MB-231 cells detected by flow cytometry.

1. * Corresponding author.

   *E-mail address*: liwentong11@163.com [↑](#footnote-ref-1)
2. * Corresponding author.

   *E-mail address*: dejunding@wfmc.edu.cn [↑](#footnote-ref-2)
